# Sustaining pneumococcal vaccination after transitioning from Gavi support: a modelling and cost-effectiveness study in Kenya

**DOI:** 10.1016/S2214-109X(18)30562-X

**Published:** 2019-04-15

**Authors:** John Ojal, Ulla Griffiths, Laura L Hammitt, Ifedayo Adetifa, Donald Akech, Collins Tabu, J Anthony G Scott, Stefan Flasche

**Affiliations:** a KEMRI-Wellcome Trust Research Programme, Centre for Geographic Medicine—Coast, Kilifi, Kenya; b Department of Infectious Disease Epidemiology, Faculty of Epidemiology and Population Health, London School of Hygiene & Tropical Medicine, London, UK; c Department of Global Health and Development, London School of Hygiene & Tropical Medicine, London, UK; d Department of International Health, Johns Hopkins Bloomberg School of Public Health, Baltimore, MD, USA; e Kenya Ministry of Health, Nairobi, Kenya; f UNICEF Health Section, Programme Division, New York, NY, USA

## Abstract

**Background:**

In 2009, Gavi, the World Bank, and donors launched the pneumococcal Advance Market Commitment, which helped countries access more affordable pneumococcal vaccines. As many low-income countries begin to reach the threshold at which countries transition from Gavi support to self-financing (3-year average gross national income per capita of US$1580), they will need to consider whether to continue pneumococcal conjugate vaccine (PCV) use at full cost or to discontinue PCV in their childhood immunisation programmes. Using Kenya as a case study, we assessed the incremental cost-effectiveness of continuing PCV use.

**Methods:**

In this modelling and cost-effectiveness study, we fitted a dynamic compartmental model of pneumococcal carriage to annual carriage prevalence surveys and invasive pneumococcal disease (IPD) incidence in Kilifi, Kenya. We predicted disease incidence and related mortality for either continuing PCV use beyond 2022, the start of Kenya's transition from Gavi support, or its discontinuation. We calculated the costs per disability-adjusted life-year (DALY) averted and associated 95% prediction intervals (PI).

**Findings:**

We predicted that if PCV use is discontinued in Kenya in 2022, overall IPD incidence will increase from 8·5 per 100 000 in 2022, to 16·2 per 100 000 per year in 2032. Continuing vaccination would prevent 14 329 (95% PI 6130–25 256) deaths and 101 513 (4386–196 674) disease cases during that time. Continuing PCV after 2022 will require an estimated additional US$15·8 million annually compared with discontinuing vaccination. We predicted that the incremental cost per DALY averted of continuing PCV would be $153 (95% PI 70–411) in 2032.

**Interpretation:**

Continuing PCV use is essential to sustain its health gains. Based on the Kenyan GDP per capita of $1445, and in comparison to other vaccines, continued PCV use at full costs is cost-effective (on the basis of the assumption that any reduction in disease will translate to a reduction in mortality). Although affordability is likely to be a concern, our findings support an expansion of the vaccine budget in Kenya.

**Funding:**

Wellcome Trust and Gavi, the Vaccine Alliance.

## Introduction

Most African countries have introduced the pneumococcal conjugate vaccine (PCV) in their childhood immunisation programmes, which has led to a substantial reduction in pneumococcal disease.^1^, ^2^ Kenya introduced the ten-valent PCV (PCV10) in 2011, with support from Gavi, the Vaccine Alliance. In Kilifi, a coastal area in Kenya with enhanced surveillance for bacterial diseases, overall invasive pneumococcal disease (IPD) decreased by 68% in the post-vaccination period (defined as 2012–16) in children younger than 5 years.^3^

Although PCVs are among the most expensive vaccines available, Gavi paid for the majority of vaccine costs when most African countries introduced PCV.^4^ However, countries are expected to transition from Gavi support and subsequently take over the full costs once their 3 year average gross national income per capita exceeds US$1580. Currently three African countries (Angola, Republic of the Congo, and Nigeria)^5^ are in the accelerated transition phase,^6^ and six more (Ghana, Côte d'Ivoire, Lesotho, Sudan, Kenya, and Zambia) are expected to join within the next 5 years.^7^, ^8^, ^9^, ^10^, ^11^, ^12^ With the increase in PCV costs at the point of transition, countries will need to independently assess the cost-effectiveness and the affordability of sustaining PCV use.

Kenya has recently entered the preparatory transition phase, which will see the country's current contribution of $0·21 per dose increase by 15% every year.^6^ In 2022, Kenya will enter the accelerated transition phase, which gradually increases the country's cost contribution to the full Gavi price of $3·05 by 2027. In 2027, PCV costs will be 15 times higher than current expenditure.^6^ Hence, before entering the accelerated transition phase, Kenya will need to evaluate whether to continue or discontinue PCV. We aimed to assess the incremental impact and cost-effectiveness of continuing PCV.

**Research in context**

**Evidence before this study**

We searched PubMed, African Journals Online (AJOL), and Cochrane Reviews databases for cost-effectiveness studies of pneumococcal conjugate vaccine (PCV) in Kenya published up to January, 2018. We excluded studies from the search results that did not report cost-effectiveness analyses of PCV in Kenya. We used the following keywords and their close variations in the search strings/logics: pneumococcus; conjugate vaccine; vaccination; economic modelling; cost-effectiveness; cost-benefit; Kenya; Africa; and Gavi.

Our search identified no previous studies reporting on the cost-effectiveness of continuing PCV after Kenya transitions from Gavi financial support. One study assessed the cost-effectiveness of the ten-valent PCV and 13-valent PCV in Kenya in the immediate (5 years) post-introduction period (Ayieko P and colleagues, 2012). However, the study did not consider whether it would be worth sustaining PCV in the period in which disease has reached a new and low-incidence equilibrium in a mature PCV era.

**Added value of this study**

Our study combined transmission and economic modelling to estimate the incremental cost-effectiveness of continuing PCV at full cost after transitioning from Gavi financial support. We found that continuing PCV use will be necessary to sustain the gains made in reducing pneumococcal disease and that it is cost-effective (based on the Kenyan gross domestic product [GDP] per capita of $1445, and compared with other vaccines). However, Kenya will need to more than double its current overall vaccine procurement budget to continue PCV use. Our findings are timely for informing upcoming recommendations by the Kenyan National Immunization Technical Advisory Group on continued PCV use in Kenya.

**Implications of all the available evidence**

Our findings suggest that to sustain the benefits of PCV, the Kenyan Government needs to make more financial commitments towards PCV procurement than it is currently spending. An intervention can be cost-effective based on common thresholds such as GDP per capita, but that is not sufficient to conclude that resources will therefore be made available. Our results should be of interest for many low-income countries finding themselves in similar deliberations but lacking similarly detailed data that we have used in our study, or the capacity to synthesise this evidence in a cost-effectiveness analysis.

## Methods

### Overview

In this study we used a dynamic pneumococcal transmission model in combination with a costing model to estimate the cost-effectiveness of the two major policy options for PCV use in Kenya from 2022—ie, the continuation of PCV use at Gavi's current and scheduled prices, or discontinuation of the vaccine. The approach accounts for the uncertainty in both epidemiology and costing estimates, and propagates it to the predicted outcomes. The study was part of the Pneumococcal Conjugate Vaccine Impact Study (PCVIS) approved by the Kenya Medical Research Institute (KEMRI) Ethical Review Committee (SSC 1433). It has an additional approval by the Oxford Tropical Research Ethics Committee (OXTREC 30-10), with delegated authority from the London School of Hygiene & Tropical Medicine (LSHTM) Research Ethics Committee.

### Disease model and incidence prediction

The details of the transmission model have been described elsewhere.^13^ In brief, we used a compartmental, age-structured, dynamic model (appendix). The model has a Susceptible-Infected-Susceptible structure for three serotype groups: the vaccine serotypes, strongly competitive non-vaccine serotypes, and weakly competitive non-vaccine serotypes. We calibrated the model to age-stratified annual pre-vaccination (2009–10) and post-vaccination (2011–16) pneumococcal carriage prevalence by fitting serotype competition, susceptibility to infection if exposed, and vaccine efficacy using non-informative priors for all parameters except the vaccine efficacy (appendix). Estimating competition parameters within the observed data increases the validity of the projections of serotype replacement disease within the model. We did not include the effect of treatment of pneumococcal disease on the prevalence of carriage in the transmission model in view of the fact that disease is very rare compared with carriage (ie, it is unlikely that treatment would affect carriage).

In January, 2011, in Kilifi, PCV vaccination was introduced together with a catch-up campaign in children younger than 5 years. To extrapolate findings to the rest of Kenya, where PCV was introduced without a catch-up campaign, the fitted model was re-run under these conditions. We predicted carriage incidence for a 15-year period, from 2017 to 2032. We predicted IPD incidence by multiplying modelled age-specific carriage incidence with case-to-carrier ratios. For each model posterior, the case-to-carrier ratios were calculated as the ratio of the observed pre-vaccination IPD incidence at Kilifi County Hospital^3^ to modelled pre-vaccination carriage incidence. The case-to-carrier ratios were assumed to remain unchanged post-vaccination. The 15-year period was chosen because it is a scope of time that the Kenyan Government generally uses for long-term health policy and strategy documents and its Vision 2030 development goals.^14^, ^15^ 15 years is also the usual length of time between the publication of results of phase 3 clinical trials and licensure and introduction of new vaccines,^16^ which means that it is unlikely that a new vaccine against *Streptococcus pneumoniae* will be licensed and introduced in Kenya during this time period.

IPD was defined as isolation of *S pneumoniae* from a sterile site culture in an individual admitted to Kilifi County Hospital. We split the predicted IPD incidence into the age-dependent proportions that are pneumococcal meningitis, pneumococcal sepsis, and bacteraemic pneumococcal pneumonia incidence on the basis of the distribution observed in clinical data from the hospital (appendix). We defined pneumococcal meningitis as isolation of *S pneumoniae* from cerebrospinal fluid (CSF) or isolation of *S pneumoniae* from blood, accompanied by a CSF white blood cell count of 50 × 10^6^ cells per L or greater or a ratio of CSF glucose to plasma glucose of less than 0·1. Bacteraemic pneumococcal pneumonia was defined as IPD with no pneumococcal meningitis but with severe or very severe pneumonia (as defined by WHO).^17^ Pneumococcal sepsis was defined as IPD not meeting the definitions of pneumococcal meningitis or bacteraemic pneumococcal pneumonia. We further assumed that for every case of IPD prevented, 4·98 cases of clinically defined pneumonia would also be prevented.^3^, ^18^ This ratio was estimated by dividing the vaccine-preventable clinical pneumonia incidence (329 per 100 000 per year)^18^ by vaccine-preventable IPD incidence (66·3 per 100 000 per year),^3^ which were both estimated from surveillance at Kilifi County Hospital. We included uncertainty in the ratio by sampling from normal distributions with means (and SDs) equal to the point estimates of vaccine-preventable IPD and clinical pneumonia incidence worked out using the observations at Kilifi County Hospital. The hospital surveillance underestimated the incidence of pneumonia and meningitis by 45% and 30%, respectively (table 1).^19^ We accounted for this syndrome-independent under-reporting in our analysis by inflating case numbers commensurately.

**Table 1:** Economic and health parameters included in the probabilistic sensitivity analysis

		**Point estimate**	**Statistical distribution**	**Source**
**Proportions of patients with access to care**				
Hospital care for sepsis, bacteraemic pneumonia, and non-bacteraemic pneumonia		55%	Beta (55,45)	Moïsi et al^19^
Hospital care for meningitis		70%	Beta (70,30)	Moïsi et al^19^
Outpatient care for IPD and non-bacteraemic pneumonia		63%	Beta (63,37)	Källander et al^20^
**Health outcomes**				
Proportion of patients with meningitis who developed sequelae		25%	Beta (25,75)	Edmond et al^21^
Case fatality rate with hospital care				
	Sepsis, bacteraemic pneumonia and meningitis: children (<15 years)	19%	Beta (19,81)	Kilifi Country Hospital
	Sepsis, bacteraemic pneumonia and meningitis: adults (≥15 years)	46%	Beta (46,54)	Kilifi Country Hospital
	Non-bacteraemic pneumonia	5·7%	Beta (6,94)	Berkley et al^22^
Case fatality rate without hospital care				
	Meningitis	97%	Beta (97,3)	Ayieko et al^23^
	Sepsis and bacteraemic pneumonia	50%	Beta (4,4)	Ayieko et al^23^
	Non-bacteraemic pneumonia	12%	Beta (12,88)	Ayieko et al^23^
**Vaccination costs (US$)**				
Vaccine price per dose		$0·21–3·05 (appendix)	Fixed	Gavi, the Vaccine Alliance^6^, ^7^, ^24^
Safety boxes		$0·46	Fixed	UNICEF^25^
Auto-disable syringes		$0·045	Fixed	UNICEF^25^
Vaccine delivery cost per dose		$1·42	Gamma (4,0·4)	Mvundura et al^26^
Syringe wastage		5%	Fixed	Ayieko et al^23^
Vaccine wastage		15%	Fixed	Gavi, the Vaccine Alliance,^23^, ^27^ Parmar et al^28^
**Treatment costs (US$)**				
With hospital care				
	Meningitis	$357·74	Gamma (4,97)	Ayieko et al^29^
	Sepsis, bacteraemic, and non-bacteraemic pneumonia	$74·64	Gamma (4,19)	Ayieko et al^29^
With outpatient care (all four disorders)		$2·74	Gamma (4,0·75)	Larson et al^30^
Without hospital care (all four disorders)		$1·15	Gamma (4,0·3)	Larson et al^30^

IPD=invasive pneumococcal disease.

### Vaccination programme costs

The programme costs included vaccine costs, vaccine wastage, safety boxes, administering syringes for each dose, syringe wastage, and vaccine delivery cost (table 1). Annual vaccine cost was calculated according to Gavi transitions rules (appendix). The vaccine delivery cost included the vaccine supply chain cost and immunisation service delivery cost. The initial investment in expanding the cold chain capacity in 2011 was not included. A switch from two-dose to four-dose presentation for Gavi countries was announced in 2017.^27^ The four-dose presentation has a preservative and once opened for the first time the vial can be kept for up to 28 days; therefore, no noteworthy change in vaccine wastage rates is expected.^27^

### Treatment costs

We adopted a societal perspective in our analyses—ie, including direct medical costs, the opportunity cost of caretaker time, and household out-of-pocket costs. To apply the appropriate treatment costs, we divided the cases into three groups depending on where individuals were treated: in hospital, as outpatients, or outside of medical care (table 1). All costs not referring to 2016 were converted into 2016 US$ for our analysis by using the International Monetary Fund's gross domestic product (GDP) deflators for Kenya.^31^

### Disability-adjusted life-years

The treatment costs for the predicted number of cases for the four diseases studied and the vaccination cost of birth cohorts were estimated and used to calculate the costs per disability-adjusted life-year (DALY) averted. We calculated the years lost due to disability as the product of disease incidence, duration of disease, and disability weights.^32^ We used disability weights from the 2013 Global Burden of Disease study^33^ in calculating the years lived with disability (YLD) component of DALYs. We used the disability weight of 0·133, assigned for infectious diseases with severe acute episodes, for both IPD and non-bacteraemic pneumonia episodes. For meningitis sequelae, we used a disability weight of 0·542 assigned for motor plus cognitive impairment. We assumed a duration of 15 days for all IPD syndromes and 7 days for non-bacteraemic pneumonia.^20^ Meningitis sequelae were assumed to last a lifetime.^21^, ^34^ We used the Kenyan age-specific life expectancies^35^ in calculating the years of life lost (YLL) due to death. The discount rate on costs and DALYs was set at 3%.

### Sensitivity analysis of the cost inputs and disease model

The full uncertainty of both epidemiological and cost parameters was propagated to the results as follows: for each posterior estimate of the epidemiological model we sampled a set of cost parameters from the pre-set distributions, effectively combining probabilistic fitting of the epidemiological model with a probabilistic sensitivity analysis of the costing model (table 1). We performed a univariable sensitivity analysis for case-fatality rates, access to hospital care rates, and the ratio of vaccine preventable clinically-defined pneumonia to vaccine preventable IPD. We estimated the impact on DALYs averted and deaths averted between 2022 and 2032 if these inputs were changed by 10% of the original value (an arbitrary choice). These variables were chosen either because of their direct correlation with mortality, which is a major contributor to DALYs, or the number of non-bacteraemic pneumococcal pneumonia cases, which forms the largest fraction of pneumococcal disease syndromes.

In Kenya, it has been recorded that children who are carriers of vaccine-type pneumococci respond less well to vaccine than non-carriers.^36^ To assess structural uncertainty in our model, we ran our analyses either with or without accounting for hyporesponsiveness. In the base case, we estimated a single vaccine efficacy independent of carrier status; in the sensitivity analysis, vaccine efficacy was estimated separately in vaccine-type carriers and in others. We also present two scenarios of discounting—ie, discounting both costs and DALYs at 3% (base case) or discounting costs alone.

### Role of the funding source

The funder of the study had no role in study design, data collection, data analysis, data interpretation, or writing of the report. The corresponding author had full access to all data used and had the final responsibility to send the manuscript for publication.

## Results

**Figure 1:**
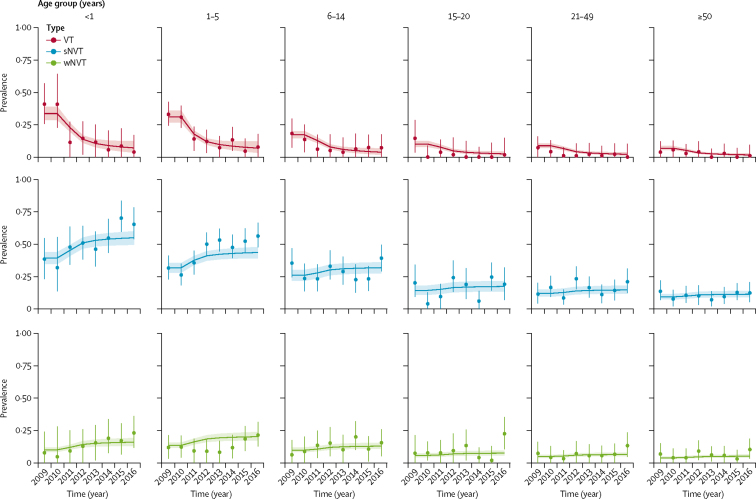
Model fit to carriage data Recorded (circles, with 95% credible intervals shown by vertical lines) and predicted (horizontal lines, with 95% predictive intervals shown by shaded areas) carriage prevalence of different vaccine serotypes over time. VT=vaccine serotypes. sNVT=strong non-vaccine serotypes. wNVT=weak non-vaccine serotypes.

**Figure 2:**
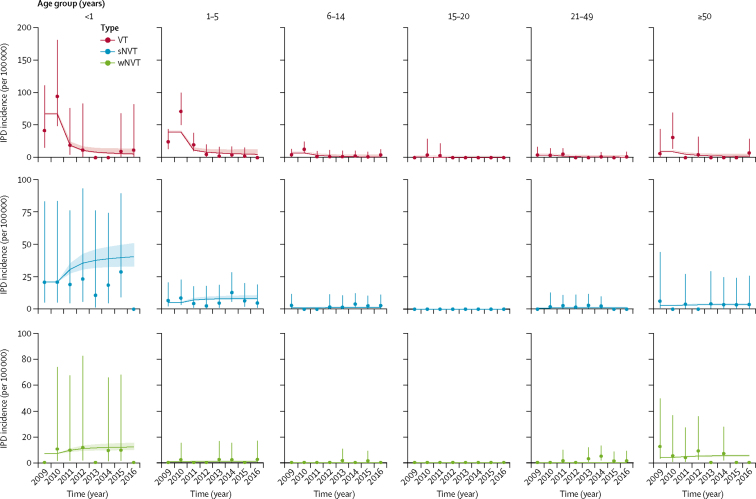
Model fit to IPD incidence data Recorded (circles, with 95% credible intervals shown by vertical lines) and predicted (horizontal lines with 95% predictive intervals shown by shaded areas) IPD incidence of vaccine serotypes over time. IPD=invasive pneumococcal disease. VT=vaccine-serotypes. sNVT=strong non-vaccine serotypes. wNVT=weak non-vaccine serotypes.

**Figure 3:**
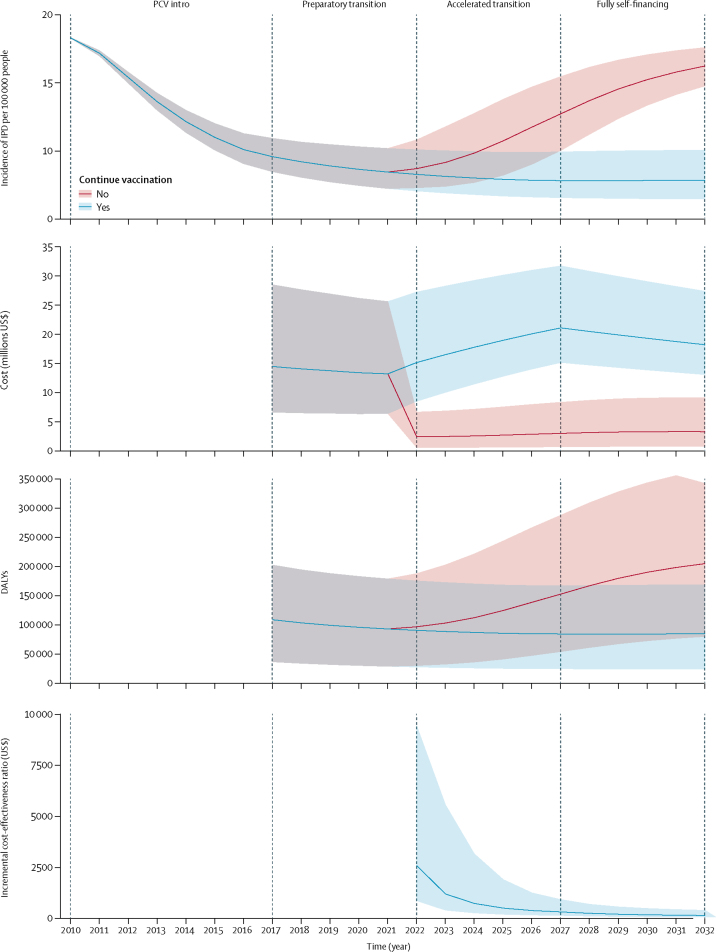
IPD incidence, costs, DALYs, and incremental cost-effectiveness ratio if PCV is continued or discontinued in Kenya, 2022–32 Shaded areas are 95% prediction intervals. Vertical dotted lines indicate Gavi transition stages. DALY=disability-adjusted life-year. IPD=invasive pneumococcal disease. PCV=pneumococcal conjugate vaccine.

There was good agreement between the observed and fitted age group and serotype-group specific carriage prevalence (figure 1, appendix) and IPD incidence (figure 2). Findings of our modelling study suggest that if cohorts of children born after the start of Jan 1, 2022 are no longer vaccinated with PCV, IPD incidence will increase from 8·5 per 100 000 in 2022 to 16·2 per 100 000 per year in 2032, equalling pre-PCV levels (figure 3). Alternatively, continuing with PCV is predicted to result in additional small reductions in IPD incidence to 7·9 per 100 000 per year in 2032, and to avert 14 329 (95% PI 6130–25 256) deaths and 101 513 (4386–196 674) IPD and non-bacteraemic pneumonia cases during the 11 years considered, as compared with discontinuing PCV in 2022.

If vaccination was stopped in 2022, the estimated average annual treatment cost for pneumococcal disease in Kenya would be $2·9 million. Otherwise, average annual treatment and vaccination costs for continuing PCV during 2022–32 were estimated as $18·7 million (95% CI 12·9–29·4; table 2). The treatment cost averted by continuing PCV from 2022 to 2032 was higher in the 1–5 year age group (49·6% of the costs averted), for those with non-bacteraemic pneumonia (65·9%), and in cases treated as inpatients (97·7%; table 3). We predicted that discontinuing PCV would partly sustain direct and indirect protection from the vaccination of previous cohorts for some of the study period, with a gradually declining effect on IPD incidence. As a result, we predict that continuation of PCV will not be cost-effective initially. However, we show that within only 1 year after the decision to continue PCV, the incremental cost-effectiveness ratio (ICER), in comparison to discontinuing PCV, improves substantially towards the threshold of the Kenyan GDP per capita ($1455 in 2016) and continues to improve throughout the study period (figure 3). Compared with discontinuing PCV in 2022, we predicted that in 2032, the cost per DALY averted would be $153, the cost per case averted $952, and the cost per death averted $6856 (table 2). The DALYs averted over the period 2022 to 2032 were higher in the 1–5 year age group (54·0% of the DALYs averted), those with non-bacteraemic pneumonia (46·9%), cases treated as outpatients (39·9%), and mortality (98·1%) compared with morbidity (table 3).

**Table 2:** Estimated costs and cost-effectiveness ratios for different scenarios

	**Average annual treatment cost, 2022–32, US$ (95% PI)**	**Cost per DALY averted in 2032, US$ (95% PI)**	**Cost per case averted in 2032, US$ (95% PI)**	**Cost per death averted in 2032, US$ (95% PI)**
Stopping vaccination in year 2022	2 898 780 (630 279–8 095 725)	Ref	Ref	Ref
Continuing vaccination	18 747 118 (12 850 031–29 392 111)	153 (70–411)	952 (302–5688)	6856 (3144–8464)
Continuing vaccination (discounting costs only)	18 747 118 (12 850 031–29 392 111)	72 (33–196)	575 (183–3442)	4148 (1901–11 171)

DALY=disability-adjusted life year. Ref=reference intervention or strategy.

**Table 3:** Treatment costs and DALYs averted by age group, disorder, type of care, and morbidity versus mortality

	**Treatment cost averted, 2022–32, US$ (95% PI)**	**DALYs averted, 2022–32, (95% PI)**
**Age group, years**		
<1	1 950 585 (306 762 to 6 022 265)	98 042 (24 860 to 210 299)
1–5	5 536 181 (916 766 to 16 387 094)	312 198 (128 126 to 565 969)
6–14	1 807 771 (454 777 to 4 694 186)	85 087 (37 529 to 149 149)
15–20	138 474 (36 385 to 351 701)	7491 (3960 to 12 241)
21–49	1 149 221 (289 609 to 2 992 496)	56 268 (29 450 to 92 727)
50+	579 816 (96 867 to 1 718 939)	18 685 (9283 to 31 832)
**Disorder**		
Meningitis	2 121 683 (617 639 to 5 255 538)	74 642 (50 769 to 96 115)
Sepsis	880 777 (274 687 to 2 119 485)	125 713 (78 008 to 185 066)
Bacteraemic pneumonia	750 696 (229 347 to 1 841 014)	107 118 (56 084 to 172 029)
Non-bacteraemic pneumonia	7 266 638 (1 119 166 to 25 663 651)	272 115 (−53 007 to 668 776)
**Type of care**		
Inpatient	10 850 030 (2 276 101 to 31 083 016)	213 734 (90 487 to 404 828)
Outpatient	212 093 (7451 to 735 443)	223 871 (88 058 to 443 001)
No hospital care	43 216 (360 to 233 991)	123 679 (24 683 to 322 589)
**Mortality versus morbidity**		
Morbidity	..	10 623 (6382 to 15 497)
Mortality	..	570 063 (241 034 to 1 012 605)

**Figure 4:**
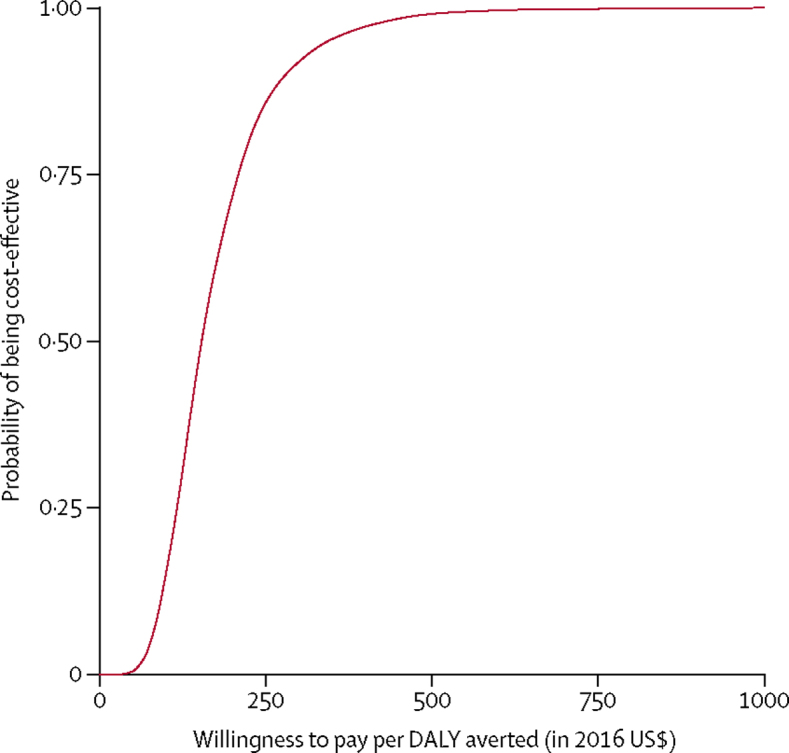
Cost-effectiveness acceptability curve Probability of continuing pneumococcal conjugate vaccine being cost-effective against the willingness to pay per DALY averted. DALY=disability adjusted life-year.

Results of sensitivity analysis showed that when using the 2016 Kenyan GDP per capita of $1455 as a threshold to determine cost effectiveness, more than 99·5% of posterior samples indicated that continuation of PCV vaccination would be cost effective for just under 6 years after 2022 (data not shown). Continuing vaccination had a 99·9% probability of being cost-effective at a willingness to pay per DALY averted of $800 (figure 4). Compared with discounting both costs and DALYs, discounting costs alone resulted in an ICER that was twice as favourable (table 2). Increasing the case fatality rates by 10% was estimated to increase the DALYs and deaths averted between 2022 and 2032 by 18 900 and 511, respectively, compared with baseline totals of 580 000 and 14 329, respectively. Increasing the proportion of cases that accessed hospital care by 10% reduced the DALYs and deaths averted by 34 800 and 870, respectively, while increasing the ratio of vaccine-preventable clinically-defined pneumonia to vaccine-preventable IPD by 10% increased the DALYs and deaths averted by 36 900 and 920, respectively, between 2022 and 2032.

We estimated that the effect of hyporesponsiveness was relatively small. Vaccine serotype carriers had a vaccine efficacy estimate against carriage that was 4 percentage points lower than that for other vaccines (appendix); as such, omitting this mechanism in the model structure led to similar results (appendix). Therefore, we did not include hyporesponsiveness in our final model.

## Discussion

Like several other low-income countries, Kenya will soon be expected to take over the full cost of national pneumococcal conjugate vaccination procurement. In this study, we have estimated the cost-effectiveness of continuing PCV using Gavi's schedule of vaccine prices, which will reach a peak at $3·05 per dose in 2027, at which point Kenya will become fully self-financing. Our model projects that discontinuing PCV would lead to an increase in IPD burden equivalent to pre-vaccination levels within 10 years. Initially, continuing vaccination might not be cost-effective because of the benefits accrued through vaccination of previous cohorts. However, the cost-effectiveness becomes substantially more favourable within a few years and, by 2032, the cost (in 2016 US$) plateaus at $153 (95% PI $70–$411) per discounted DALY averted.

The most commonly used threshold for judging the cost-effectiveness of an intervention is a country's GDP per capita. Using this criterion, we find continuation of PCV in Kenya after transition from Gavi support highly cost-effective. The GDP per capita threshold was initially supported by the Commission on Macroeconomics and Health^37^ and adopted by WHO's Choosing Interventions that are Cost-Effective project (WHO-CHOICE). The use of GDP-based thresholds has been criticised for four main reasons: it does not consider the cost–benefit profile of interventions competing for the same health budget; it does not adequately address the willingness to pay; it does not address affordability; and it is too easily attained.^38^ Alternatives include benchmarking of interventions by assessing a country's willingness to pay by comparing cost-effectiveness ratios against that of vaccines currently in use.^38^

The cumulative costs per DALY averted of introducing the Rotarix or the RotaTeq rotavirus vaccines in Kenya have been estimated as $200 and $406 (2016 US$), respectively.^39^ Similar to our estimates, these were derived on the basis of a societal perspective with a 3% discounting of both costs and benefits.^39^ The *Haemophilus influenzae* type b (Hib) vaccine was introduced in 2001 in Kenya as part of the pentavalent vaccine.^40^ In a static model developed to follow the Kenyan 2004 birth cohort until death, with and without Hib vaccine, it was estimated that the discounted (3% for both costs and benefits) cost per DALY averted of introducing Hib vaccine was $85 (2016 US$) from a health provider perspective.^41^ This calculation suggests that continuation of PCV is less cost-effective than the Hib vaccine and more cost-effective than the rotavirus vaccine. However, these comparisons must be tempered by the fact that the rotavirus analysis ignored herd immunity, while the Hib analysis took a health provider perspective, both of which decrease cost-effectiveness.

However, cost-effectiveness does not necessarily imply affordability. The latter depends on available resources in the health budget, or any other sources within the national accounts that can fill the gap in the health budget. Budgetary allocation to the health sector as a fraction of national government budget has slightly decreased from 4% in the financial year 2014–15 to 3·7% in the financial year 2016–17.^42^ The Kenyan annual health budget for 2015 was $600 million.^42^ Of this, $6·9 million (0·8%)^43^ was spent on vaccines. This has been possible because Kenya only needs to fund 10% of its vaccines from its revenues; donors fund the rest of the budget.^7^ We have estimated that continuing with PCV after 2022 will require an additional $15·8 million annually compared with discontinuing PCV; in other words, it will more than double Kenya's current expenditure on vaccines. At the same time, following transition from Gavi support, the Kenyan Government's financial contribution for pentavalent, rotavirus, and yellow fever vaccines will need to increase as well if Kenya wants to sustain its current vaccine portfolio.

Several initiatives indicate that the cost of PCV procurement might be reduced in the future. For instance, the Serum Institute of India is developing a ten-valent PCV with a target per-dose price of $2·00.^44^ Also, in settings where vaccine serotypes have been eliminated from circulation, it might be possible to sustain control of transmission using a two-dose or even one-dose schedule.^45^ If vaccine serotypes can be eliminated in Kenya—for example by additional efforts such as a catch-up campaign—then the shift to a reduced dose schedule might also be feasible. By 2022, most of these options will have a wider evidence base that might allow their formal consideration. Currently there is insufficient support to include them in our analyses, but if proven to be effective, these aspects will further improve on our PCV cost-effectiveness estimates of sustaining PCV in Kenya.

There are potential limitations to our study. The proportion of individuals with pneumococcal disease treated in hospital, as outpatients, or those who do not access care is a key determinant of both costs incurred and DALYs, by determining the case fatality rate. Overestimating the proportion of cases that get hospital treatment would mean that the overall costs of treatment were overestimated while the fatal cases, and therefore DALYs, were underestimated. The overall effect would be an overestimated ICER, which is conservative. In our analysis, we estimated the proportion of cases that were treated in hospital using local surveillance data. However, we did not have local information about what proportion among non-hospitalised cases are treated as outpatients; this was obtained from a Ugandan verbal autopsy study among fatal pneumonia cases.^20^ It is possible, therefore, that we have overestimated the number of non-hospitalised patients treated as outpatients, and, by extension, overestimated the ICER. The case-fatality rate for disease cases that do not access hospital care was obtained from a study^23^ that had used expert opinion for the measure. The lack of data on this input persists. We have estimated that a 10% increase or decrease in case-fatality rates leads to an increase or decrease in DALYs and deaths averted between 2022 and 2032 by 18 900 and 511, compared with baseline totals of 580 000 and 14 329, respectively; these changes are about 3·3% and 3·6% of the baseline totals, respectively.

In our study, carriage prevalence used in model fitting and IPD incidence data were obtained from Kilifi, but we made inferences about the rest of the country. This raises the question: how representative is Kilifi of the rest of Kenya in terms of health and health system indicators? Using under-5 mortality as a crude index of health, Kilifi does not stand out, with a rate of 72 per 1000 livebirths compared with the national estimates of 79 per 1000.^46^ The national mean annual rate of cases hospitalised with severe acute respiratory illness of 228·1 (95% CI 208·1–249·4) per 100 000 people is similar to that of the coastal region, 213·3 (193·5–234·7) per 100 000 people, which covers Kilifi.^47^ In a service availability and readiness assessment report for Kenya,^48^ the mean availability of services for eliminating communicable conditions was 54% nationwide and 51% in Kilifi county. The national health facility density per 10 000 population was 2·04 compared with 1·99 in Kilifi county.^48^ In Kenya, the annual number of visits to health-care providers per person was 3·1, and 12·7% of people reported sickness but did not seek health care; in Kilifi the estimates were 3·0 and 10·8%, respectively.^49^

We included vaccine impact on clinically defined pneumonia but not on otitis media and sinusitis; hence our estimates of impact are conservative. We extrapolated the impact of PCV10 on clinically-defined pneumonia from its effect on IPD. We estimated the ratio of the vaccine-preventable disease incidence (VPDI) for IPD to the VPDI for clinically-defined pneumonia from surveillance data in Kilifi, Kenya, following PCV10 introduction.^18^ Our estimate (4·98, 95% UI 0·27–8·91) is lower than the ratio of IPD to pneumonia in other settings. For example, radiologically-confirmed pneumonia was estimated to be 7·5 times more common than IPD in a clinical trial in The Gambia;^50^ all-cause pneumonia was estimated to be 216 times more common than IPD in the USA.^51^ Much higher values for this ratio are probably a reflection of a lower prevalence of pneumococcal disease among the chosen case definition, leading to a lower positive predictive value for pneumococcal aetiology. The internal validity of our own estimate is underpinned by the fact that it was derived using the same surveillance setting for both IPD and clinically-defined pneumonia.

Cost-effectiveness analyses are strongly affected by the effect of the intervention on mortality and yet data on mortality impacts are relatively sparse, especially in countries that lack cause-specific vital registration systems such as Kenya. We estimated the mortality impact of PCV10 by multiplying the incidence of cases with different pneumococcal syndromes by the case fatality ratio for that syndrome. The case fatality ratios were adjusted according to whether the patient received inpatient care. Therefore, our cost-effectiveness analysis is largely based on the assumption that reduction in disease will translate to reduction in mortality. The rationale for using a vaccine with limited serotype coverage is that non-vaccine serotypes, which can cause serotype replacement disease, will have a lower case fatality ratio than the vaccine serotypes that they replace. If so, then our approach, which uses a constant case fatality ratio, will provide a conservative estimate of cost-effectiveness.

Findings of a meta-analysis of case fatality ratios for pneumococcal pneumonia showed that patients with pneumonia caused by PCV10 serotypes 6B and 19F and non-vaccine serotypes 3, 6A, and 9N were significantly more likely to die than patients with serotype 14 (the reference serotype in the study) and that patients infected with vaccine serotypes 1 and 7F and non-vaccine serotype 8 were significantly less likely to die.^52^ Serotypes 3 and 6A have been recorded in carriage in Kenya post vaccination,^53^ but have not been associated with increased IPD in the period.^3^ Hence, there was no clear evidence from the meta-analysis as to whether non-vaccine serotypes as a group have a higher case fatality ratio than vaccine-serotypes because the analysis was serotype-specific. If the case fatality ratio of non-vaccine serotypes is higher than vaccine serotypes, this would abrogate any mortality benefits attributable to the effect of PCV10 on vaccine serotype disease. However, in the UK, IPD-related mortality rate in children younger than 5 years decreased by 69% following PCV7 and PCV13 introduction,^54^ despite documented rapid serotype replacement in disease.^55^

In conclusion, several low-income countries will soon be transitioning out of Gavi support and will need to decide whether to sustain their pneumococcal conjugate vaccination. We show, using Kenya as an example, how ongoing detailed surveillance can be combined with mathematical modelling and health economics to inform an upcoming decision of a country's National Immunization Technical Advisory Group on the cost-effectiveness of different policy options. We estimate that maintaining PCV is essential to sustain the decreased burden of pneumococcal disease and that it is cost-effective against conventional criteria. For Kenya, affording PCV vaccination in the post-Gavi era will necessitate the country substantially increasing the proportion of health spending on routine immunisation.
