# Supplementary material for: Sustaining pneumococcal vaccination after transitioning from Gavi support: a modelling and cost-effectiveness study in Kenya
Source: Lancet Glob Health. 2019 Apr 15;7(5):e644–54. doi: 10.1016/S2214-109X(18)30562-X (PMC6484775; doi:10.1016/S2214-109X(18)30562-X)
Supplement: Supplementary appendix [file mmc1.pdf]

THE LANCET
Global Health

Supplementary appendix

This appendix formed part of the original submission and has been peer reviewed. We post it as supplied by the authors.

Supplement to: Ojal J, Griffiths U, Hammitt LL, et al. Sustaining pneumococcal vaccination after transitioning from Gavi support: a modelling and cost-effectiveness study in Kenya. *Lancet Glob Health* 2019; **7:** e644–54.

Appendix: Model structure and parameters estimates

Model structure

A more detailed description of the model and the likelihood function is presented in[1]. The brief description provided in this appendix is to help in the understanding of the notation used, without necessarily referring to[1]. The model is compartmental, age-structured and dynamic. Compartments are defined according to pneumococcal carriage states (Supplementary Figure 2). It has a Susceptible-Infected-Susceptible (SIS) structure for three serotype groups: the PCV10 serotypes, strong NVT and weak NVT.

At any point in time, an unvaccinated individual can be susceptible (non-carrying) in state S; carry a VT, V; carry a weak NVT, N_w; carry a strong NVT, N_s; carry simultaneously a weak and strong NVT, N_{sw}; carry simultaneously a VT and weak NVT, B_w; or carry simultaneously a VT and a strong NVT, B_s. Once vaccinated, the individual moves to one of the corresponding states ($S^{(v)}$, $V^{(v)}$,$N_w^{(v)}$, $N_s^{(v)}$, $N_{sw}^{(v)}$, $B_w^{(v)}$, $B_s^{(v)}$). We also fitted a model in which the efficacy of the vaccine on carriage acquisition is reduced due to prevailing carriage at the point of vaccination (hyporesponsiveness) is considered. Under this model, upon vaccination, individuals not carrying vaccine serotypes move to the corresponding states ($S^{(v)}$,$N_w^{(v)}$, $N_s^{(v)}$, $N_{sw}^{(v)}$) while those carrying vaccine serotypes ($V, B_w^{(v)}$, $B_s^{(v)}$) move to the corresponding hyporesponse-related states ($hV, hB_w^{(v)}$, $hB_s^{(v)}$) (Supplementary Figure 3).

Parameterisation

A susceptible unvaccinated individual in age group i becomes colonised with VTs, strong NVTs or weak NVTs at age-group-specific time-dependent rates (forces of infection) denoted by $\lambda_{Vi}(t)$, $\lambda_{Nsi}(t)$ and $\lambda_{Nwi}(t)$, respectively. The forces of infection were expressed as functions of the social mixing matrix and age-group specific factors (q_i) that scale the rate of social contacts into infectious contacts. Due to competition between serotypes in colonising the nasopharynx, the acquisition rate of a secondary serotype is lower than the acquisition rate

of that serotype in a completely susceptible individual. Three competition parameters, c_{v0}, c_{w0} and c_{s0}, represent the fraction by which acquisition rates of secondary serotypes are reduced in <6 year olds infected with VTs, weak NVTs and strong NVTs, respectively. Two competition parameters, $c_{vw} = c_v = c_w$ and c_s, were used for individuals aged ≥ 6 years infected with VTs/weak NVTs and strong NVTs, respectively. In the vaccinated compartments the rate of acquisition of VTs are reduced by the vaccine efficacy against carriage acquisition denoted ε, or ε_h according to whether the compartment is associated with hyporesponsiveness.

The Metropolis-Hastings algorithm was used to draw samples from the posterior distributions of the parameters. Uniform priors in the range 0-1 were used for competition parameters and the social contact scaling parameters (q_i). For the vaccine efficacy parameters we used a normal prior centered around 50% with 95% uncertainty interval of 40-60%. 50,000 adaptive MCMC iterations were used. After a burn-in of 25,000 was discarded the remaining stationary samples were thinned to 5000 to estimate the posterior distribution. Convergence was assessed graphically, by observing was no negative or positive trend (zero gradient) in the chain, and by using Geweke diagnostic to check if a chain was stationary. The thinned posterior samples of the parameters were summarised to obtain point estimates (posterior mean) and probability (credibility) intervals. The parameter estimates are shown in Table A1.

[1] Ojal J, Flasche S, Hammitt LL, Akech D, Kiti MC, Kamau T, et al. *Sustained reduction in vaccine-type invasive pneumococcal disease despite waning effects of a catch-up campaign in Kilifi, Kenya: a mathematical model based on pre-vaccination data.* Vaccine. 2017;35:4561–8.

Table A1. Estimated parameters of the dynamic transmission models

**Parameter**	**Estimate (95% Credible Interval)**	**Estimate accounting for hyporesponsiveness (95% Credible Interval)**
Competition parameters	c_{s0} = 0.42 (0.24, 0.62)<br>c_{w0} = 0.73 (0.44, 0.97)<br>c_{v0} = 0.44 (0.25, 0.70)<br>c_s = 0.11 (0.01, 0.40)<br>$c_{vw} = c_v = c_w$ = 0.70 (0.30, 0.98)	c_{s0} = 0.41 (0.25, 0.59)<br>c_{w0} = 0.70 (0.43, 0.97)<br>c_{v0} = 0.46 (0.27, 0.70)<br>c_s = 0.10 (0.01, 0.30)<br>$c_{vw} = c_v = c_w$ = 0.66 (0.24, 0.98)
Probability of infection per 100 contacts	q_1 = 0.14 (0.11, 0.19)<br>q_2 = 0.45 (0.38, 0.55)<br>q_3 = 0.30 (0.26, 0.35)<br>q_4 = 0.08 (0.06, 0.11)<br>q_5 = 0.16 (0.13, 0.19)<br>q_6 = 0.06 (0.05, 0.07)	q_1 =0.14 (0.11, 0.19)<br>q_2 =0.45 (0.39, 0.54)<br>q_3 =0.30 (0.26, 0.35)<br>q_4 =0.08 (0.06, 0.10)<br>q_5 =0.16 (0.13, 0.19)<br>q_6 =0.06 (0.05, 0.07)
Vaccine efficacy again carriage	ε = 0.59 (0.49, 0.68)	ε = 0.58 (0.47, 0.68)
Vaccine efficacy against carriage for VT carriers (hyporesponsiveness)	N/A	ε_h = 0.54 (0.40, 0.68)
